# Role of Damage DNA-Binding Protein 1 in Pancreatic Cancer Progression and Chemoresistance

**DOI:** 10.3390/cancers11121998

**Published:** 2019-12-12

**Authors:** Yiyin Zhang, Yubin Lei, Jin Xu, Jie Hua, Bo Zhang, Jiang Liu, Chen Liang, Qingcai Meng, Xianjun Yu, Si Shi

**Affiliations:** 1 Department of Pancreatic Surgery, Fudan University Shanghai Cancer Center, No. 270 Dong’An Road, Shanghai 200032, China; zhangyiyin@fudanpci.org (Y.Z.); leiyubin@fudanpci.org (Y.L.); xujin@fudanpci.org (J.X.); huajie@fudanpci.org (J.H.); zhangbo@fudanpci.org (B.Z.); liujiang@fudanpci.org (J.L.); liangchen@fudanpci.org (C.L.); mengqingcai@fudanpci.org (Q.M.); 2 Department of Oncology, Shanghai Medical College, Fudan University, No. 270 Dong’An Road, Shanghai 200032, China; 3 Shanghai Pancreatic Cancer Institute, No. 270 Dong’An Road, Shanghai 200032, China; 4 Pancreatic Cancer Institute, Fudan University, No. 270 Dong’An Road, Shanghai 200032, China

**Keywords:** pancreatic adenocarcinoma, damaged DNA-binding protein 1, chemoresistance

## Abstract

Damaged DNA-binding protein 1 (DDB1) recruits nucleotide excision pathway proteins to form the UV-damaged DNA-binding protein complex and is required for DNA repair. DDB1 was reported to participate in apoptosis and chemoresistance regulation in several cancers. However, little is known about the function of DDB1 in pancreatic adenocarcinoma (PDAC). In this study, we reported that DDB1 functions as a tumor-promoting factor in PDAC by regulating cancer cell proliferation, epithelial-mesenchymal transition (EMT) and chemoresistance. Compared to normal pancreatic tissues, PDAC tissues had high expression levels of DDB1, and this high expression was positively correlated with poor prognosis. Furthermore, reductions in cell proliferation and EMT were observed in DDB1-deficient PDAC cell lines. Intriguingly, we also found that abrogation of DDB1 expression increased PDAC cell sensitivity to gemcitabine (GEM). Mechanistically, DDB1 knockdown was associated with an increase in deoxycytidine kinase expression in vivo and in vitro. In summary, our work demonstrated that DDB1 promotes PDAC progression and chemoresistance and may serve as a potential predictive marker and therapeutic target for PDAC treatment.

## 1. Introduction

Despite the low incidence of pancreatic cancer, it still ranks as the fourth leading cause of cancer-related deaths in the United States [1]. Approximately 80% of patients have metastatic disease when first diagnosed, and other than direct surgical resection, chemotherapy remains the main treatment for pancreatic ductal adenocarcinoma (PDAC). Gemcitabine (GEM), has been regarded as the cornerstone of chemotherapy for PDAC since 1997 and various GEM-based chemotherapy combinations have been developed in the past 2 decades [2,3]. However, the efficacy of either GEM monotherapy or combinations remains disappointing. Therefore, it is of vital importance to explore the potential mechanism of GEM resistance and to invent new therapeutic strategies to conquer PDAC.

GEM enters the cytosol mainly through the human equilibrative nucleoside transporter-1 [4] and is phosphorylated into difluorodeoxycytidine monophosphate (dFdCMP) by deoxycytidine kinase (dCK). dCK converts dFdCMP into its active metabolites, which interrupt DNA synthesis by blocking the production of deoxynucleotide triphosphates by inhibiting ribonucleotide reductase subunit (RRM)1 and RRM2 [5,6]. In brief, dCK is indispensable for GEM to transform from proto-drug to its active form, and dCK expression is also correlated with patient prognosis [7,8].

Epithelial-mesenchymal transition (EMT) is critical for tumor formation, dissemination and chemoresistance in PDAC. It has been reported that tagged pancreatic epithelial cells in a PDAC mouse model maintain a mesenchymal phenotype and exhibit stem cell properties [9]. EMT status was significantly correlated with CA19-9 levels, portal vein invasion and lymph node metastasis in PDAC. Moreover, mesenchymal tumors are associated with poorer prognosis than epithelial tumors (13.7 months vs. 40.2 months) [10]. Inhibition of SNAI1 reduced the number of tumor-bearing mice and increased the membrane staining of E-cadherin, which supports that EMT is a key step towards cancer progression and metastasis in PDAC [11].

Damaged DNA-binding protein 1 (DDB1) is essential for DNA repair, and it usually binds to DDB2 to form the UV-damaged DNA-binding protein and to recruit proteins of the nucleotide excision pathway to start DNA repair [12]. The deletion of DDB1 abrogates the self-renewing capacity of hepatocytes and results in the compensatory proliferation of DDB1-expressing hepatocytes, thus leading to hepatocellular carcinoma [13]. Liu et al. found that thyroid transcription factor 1 could interact with DDB1 and block its binding to checkpoint kinase 1 (CHK1), which attenuated the ubiquitylation, subsequently inducing CHK1 degradation and contributing to lung adenocarcinoma development [14]. Furthermore, a recent study suggested that DDB1 and Cullin-RING ubiquitin ligases (CRL) 4, the ubiquitin ligase of Cullin 4A (CUL4A)-DDB1 E3, are important factors in ovarian cancer chemoresistance because they regulate apoptosis and might be therapeutic targets for patients after cisplatin failure [15]. However, the relationship between DDB1 and PDAC is not clear, and the underlying mechanism of the role that DDB1 plays in PDAC development requires further exploration.

In this study, we explored the function of DDB1 in PDAC and its potential role in the process of GEM resistance. DDB1 upregulation was associated with poor survival in PDAC patients. Our novel findings showed that DDB1 could regulate PDAC cell proliferation, apoptosis, and EMT and could sensitize cells to GEM treatment, which may be partially due to its regulation of dCK. Our study provided new insight into the predictive value of DDB1 and revealed treatment targets that show promising effects for improving the prognosis of PDAC.

## 2. Results

### 2.1. DDB1 Expression Is Positively Correlated with PDAC Prognosis

**Figure 1:**
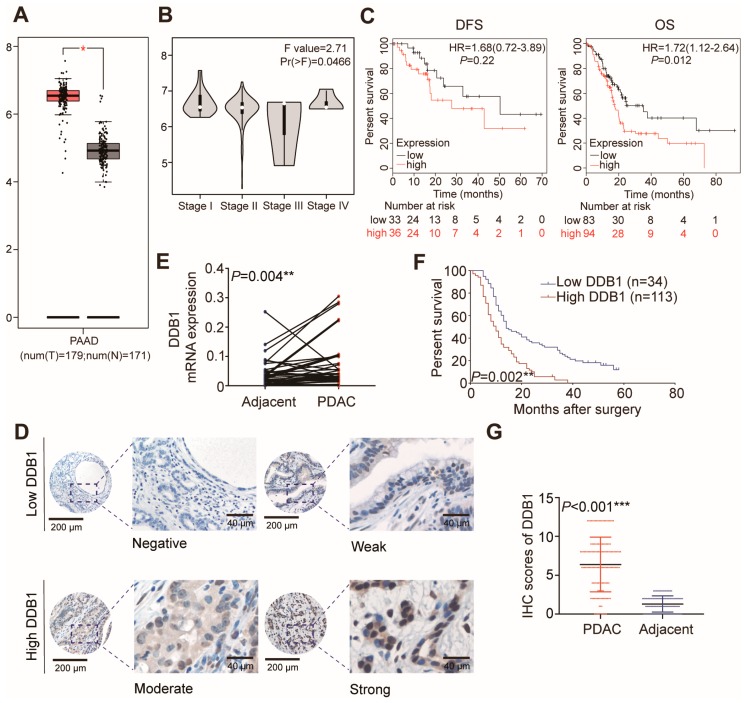
DDB1 expression is increased in PDAC tissues. (**A**) *DDB1* transcription was increased significantly in pancreatic cancer tissues compared to that in normal tissues in the GEPIA dataset. (**B**) *DDB1* transcription was varied in different stages in the GEPIA dataset. (**C**) High mRNA expression of *DDB1* was associated with shorter OS (*p* = 0.012) but not DFS (*p* = 0.22). (**D**) Representative images of IHC staining for DDB1 in TMAs (inset scale bar, 40 µm). (**E**) *DDB1* mRNA expression levels in PDAC and adjacent normal tissues (*n* = 45, *p* = 0.004). (**F**) The OS of patients with PDAC was assessed using a Kaplan-Meier analysis based on DDB1 expression (*n* = 147, *p* = 0.002). (G) DDB1 expression in PDAC and adjacent normal tissues, as determined by the IHC score (*n* = 147, *p* < 0.001).

By analyzing the publicly assessable data for of PDAC within the Gene Expression Profiling Interactive Analysis (GEPIA) dataset, we found that *DDB1* transcription was increased significantly in pancreatic cancer tissues and varied in different stages (Figure 1A,B); high mRNA expression of *DDB1* was associated with shorter overall survival (OS) (*p* = 0.012) but not disease-free survival (DFS) (*p* = 0.22; Figure 1C). This was consistent with the prognostic data from our center, as higher expression of *DDB1* was detected in tumoral areas (Figure 1E), which was confirmed at the mRNA level from 45 paired samples. We later performed immunohistochemistry (IHC) on tissue microarrays (TMAs) containing samples from 147 patients (Figure 1D). Decreased DDB1 expression was detected in adjacent tissues compared to tumoral tissues based on the IHC score (Figure 1G). The clinical characteristics of PDAC patients are presented in Table 1. High DDB1 expression was associated with a poorer median survival of 11.5 months, which was 10.1 months shorter than that of patients with low expression (Figure 1F; *p* = 0.002). According to multivariate Cox regression analysis, DDB1 was an independent prognostic marker of PDAC (Table 2).

### 2.2. DDB1 Is Required for Cell Proliferation and EMT in PDAC

**Figure 2:**
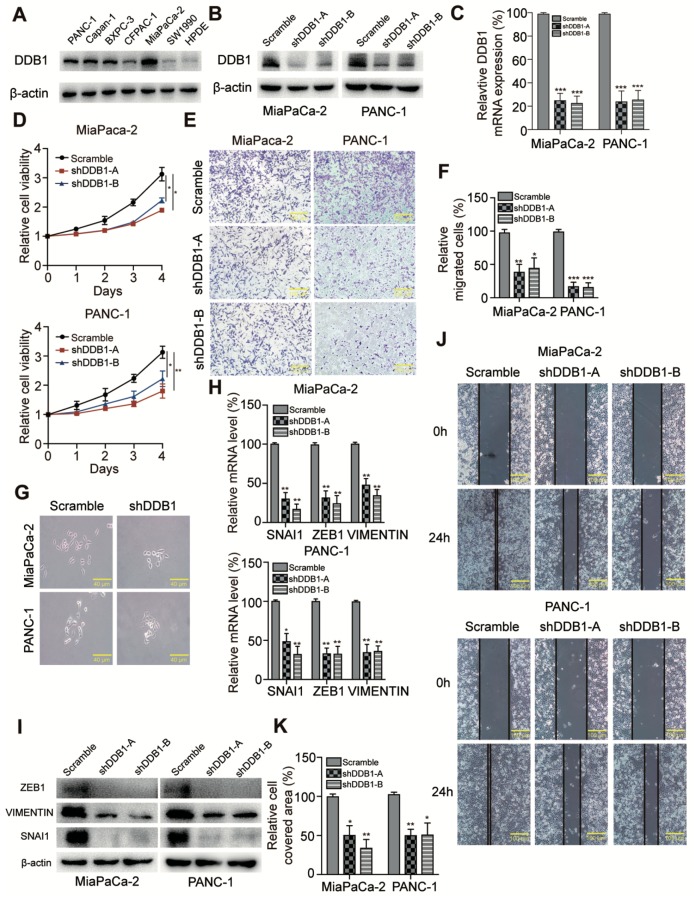
DDB1 is required for cell proliferation and EMT in PDAC. (**A**) Western blotting analysis of DDB1 expression in PDAC and the HPDE cells; β-actin was used as a control. Detailed information of Western blotting figures can be found at Supplement material Figure S1 and Table S3. (**B**) Analysis of DDB1 protein expression using a Western blotting assay; also see Supplement material Figure S1 and Table S3. (**C**) Analysis of relative gene expression data for *DDB1* using qRT-PCR. (**D**) A CCK-8 assay was used to detect the proliferation of PDAC cells transfected with DDB1 shRNA. (**E**) Cell migration analysis following DDB1 knockdown; quantitation of the data is shown in (**F**). (**G**) Morphology of PDAC cells transfected with scrambled shRNA and DDB1 shRNA (scale bar, 40 μm). (**H**) The *SNAI1*, *ZEB1* and *VIMENTIN* mRNA levels in PDAC cells were determined following DDB1 silencing and compared with those in control cells (* *p* < 0.05, ** *p* < 0.01, *** *p* < 0.001). (**I**) The expression of EMT phenotype markers was determined by Western blotting; also see Supplement material Figure S1 and Table S3. (**J**) DDB1-silenced MiaPaCa-2 and PANC-1 cells both exhibited significantly decreased cell motility in the wound healing assay; quantitation of the data is shown in (**K**).

**Figure 3:**
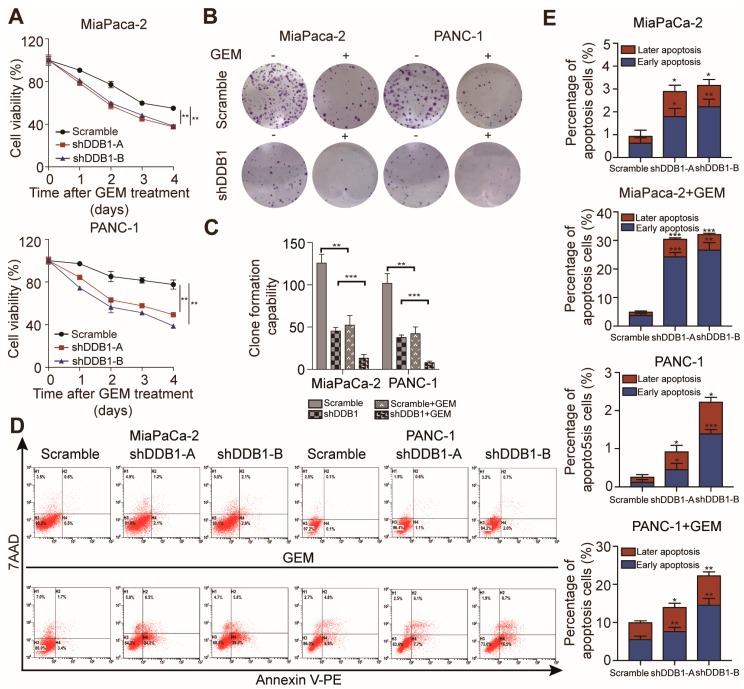
DDB1 knockdown increases the sensitivity of PDAC cells to GEM. (**A**) MiaPaCa-2 and PANC-1 cells were treated with GEM for 0–4 days. (**B**) A colony formation assay was conducted to confirm the effect of DDB1 abrogation and the effect of GEM on PDAC cell lines; quantitation of the data is shown in (**C**). (**D**) Apoptosis rates of the DDB1-silenced cell lines with or without GEM treatment; quantitation of the data is shown in (**E**). * *p* < 0.05, ** *p* < 0.01, *** *p* < 0.001.

We examined cultured cancer cell lines and observed high DDB1 expression in multiple PDAC cell lines compared with human pancreatic ductal epithelial (HPDE) cells (Figure 2A). Based on the potential oncogenic functions of DDB1 in PDAC, we hypothesized that DDB1 might influence cancer cell proliferation, migration, colony formation or apoptosis. To validate this possibility, stable DDB1-silenced cell lines were established, and knockdown efficiency was examined by Western blotting and quantitative real-time PCR (qRT-PCR, Figure 2B,C). We observed that cell proliferation was dramatically inhibited in DDB1 knockdown cell lines (Figure 2D). Furthermore, the colony formation assay reflected a reduction in the colony formation ability of DDB1-silenced cells (Figure 3B), suggesting that DDB1 possessed a vital function in PDAC cell proliferation and tumorigenesis. Intriguingly, we also observed the reversion of the EMT-like phenotype upon examining the cell morphology of DDB1-silenced PDAC cells (Figure 2G), implying that DDB1 may play a role in regulating EMT in cancer cells. To verify whether DDB1 is involved in PDAC cell motility, we performed wound healing and transwell assays in control and DDB1 knockdown cells. DDB1-silenced MiaPaCa-2 and PANC-1 cells both exhibited significantly decreased numbers of migrating cells in the transwell assays (100% vs. 37% and 43%, *p*<0.001) in MiaPaCa-2 and (100% vs. 21% and 16%, *p*<0.001) PANC-1 cells (Figure 2E,F). Consistently, the results also revealed that DDB1 silencing significantly inhibited the extent of wound closure (Figure 2J,K). As *SNAI1*, *ZEB1* and *VIMENTIN* are known biomarkers for EMT, we determined their expression by immunostaining and qRT-PCR analyses. Consistent with the cellular phenotype, DDB1 knockdown was associated with decreased SNAI1, ZEB1 and VIMENTIN expression at both the mRNA and protein levels (Figure 2H,I), indicating that DDB1 knockdown was inversely correlated with an EMT phenotype in PDAC cells.

### 2.3. Abrogation of DDB1 Expression Increases PDAC Cell Sensitivity to GEM

As EMT-like phenotypes are well known for their relationship with chemoresistance, we hypothesized that the abrogation of DDB1 expression might sensitize PDAC cells to GEM treatment [16]. We tested this by using a cell viability assay to analyze whether silencing DDB1 would affect GEM treatment. The concentration used to inhibit cell viability to 50% (IC_50_ value) was dramatically decreased in DDB1-silenced PDAC cells compared to scrambled-shRNA PDAC cells (Supplement material Table S1). Decreased cell viability and colony formation capacity were observed in DDB1 knockdown cells (Figure 3A,B). A colony formation assay also confirmed that silencing DDB1 could further reduce the colony formation ability of GEM-treated MiaPaCa-2 and PANC-1 cells compared to control cells (Figure 3B,C). Since GEM has antitumor effects mainly through inducing apoptosis, we further measured the apoptosis rate in scrambled-shRNA and DDB1-silenced PDAC cells with and without GEM treatment. Surprisingly, we found that GEM-induced apoptosis was increased in DDB1-silenced PDAC cells compared with scrambled-shRNA cells (Figure 3D,E). These results suggest that DDB1 has a vital function in GEM resistance.

### 2.4. DDB1 Knockdown Sensitizes Pancreatic Xenograft Tumors to GEM Treatment

**Figure 4:**
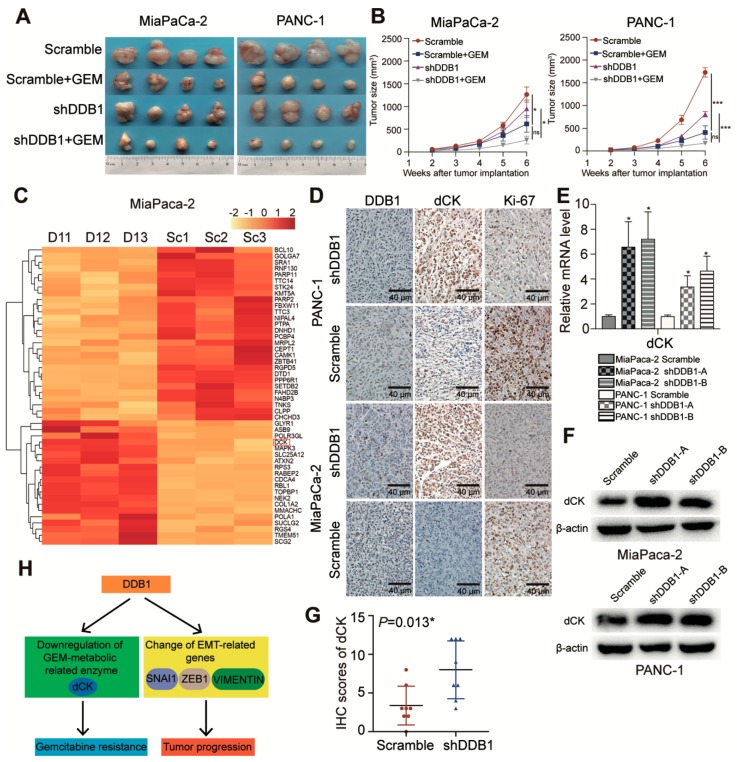
DDB1 increases the resistance of pancreatic xenograft tumors to GEM. (**A**) Stably transfected DDB1 shRNA or scrambled-shRNA PDAC cells were injected into nude mice, which were later divided into scramble, shDDB1, scramble+GEM and shDDB1+GEM groups and treated as described in the Methods. (**B**) The tumor sizes were measured by electronic Vernier calipers. Tumor growth curves were created based on the tumor volume. (**C**) The heatmap showed differentially expressed genes in shDDB1-transfected (D1) cells and scrambled-transfected (Sc) cells. (**D**) The expression of DDB1, dCK and Ki-67 was detected in tumor tissue slices from the xenografts using IHC (scale bar, 40 μm). (**E**) mRNA expression level of *dCK* in DDB1-silenced or scrambled PDAC cell lines. (**F**) Effect of DDB1 abrogation on the expression of dCK according to a Western blotting assay. Detailed information of Western blotting figures can be found at Supplement material Figure S1 and Table S3. (**G**) The correlation between DDB1 and dCK was determined by IHC scores. (**H**) The possible mechanism of DDB1-mediated GEM resistance and tumor progression due to dCK downregulation and changes in EMT-related genes in patients with PDAC. * *p* < 0.05, *** *p* < 0.001.

To further determine the inhibitory function of DDB1 in cancer cell sensitivity to GEM treatment, xenograft tumors were established. In vivo data confirmed the increased sensitivity to GEM treatment in DDB1 knockdown PDAC cells. As shown in Figure 4A,B, the tumor growth rate and size were relatively decreased in both the MiaPaCa-2 and PANC-1 cell shDDB1 groups treated with PBS compared to the control groups. Compared to the control groups, the shDDB1 groups also showed enhanced sensitivity to GEM treatment. More importantly, Ki-67 expression was reduced in shDDB1 groups treated with PBS and was remarkably reduced in shDDB1 groups treated with GEM; these results indicated a decreased cell proliferation rate in the shDDB1 groups, especially when sensitized by GEM (Figure 4D). These data demonstrated in vivo that DDB1 is essential for PDAC cells resistance to GEM treatment.

### 2.5. DDB1 Is Correlated with dCK Expression

We next explored the possible mechanism underlying GEM sensitization in DDB1-silenced PDAC cells. Through RNA sequencing, the expression of the GEM metabolic-related enzyme *dCK* was found to be associated with DDB1 in MiaPaCa-2 cells (Figure 4C). GEM requires catalysis by dCK and other enzymes to complete its transformation and activation. Low expression of dCK was reported to be associated with GEM-resistant cells [17]. Therefore, we suspected that dCK may mediate the increased sensitivity of PDAC cells to GEM treatment caused by DDB1 knockdown. Indeed, our qRT-PCR and Western blotting results showed dCK upregulation after DDB1 deletion in the MiaPaCa-2 and PANC-1 cell lines (Figure 4E,F).

We also performed IHC to determine the expression of dCK in the xenograft tumor tissue sections. As expected, dCK expression was inversely associated with DDB1 expression (*p* = 0.013; Figure 4D,G), further indicating that dCK was the effector of DDB1 in cancer cell GEM resistance. Figure 4H shows how DDB1 influenced GEM metabolism through dCK and caused the EMT phenotypes.

## 3. Discussion

Despite the improvement in diagnostic tools and the emergence of novel therapeutic agents, PDAC remains mostly unresectable when first diagnosed due to unspecific symptoms. The majority of patients who receive adjuvant therapies usually consider GEM and GEM-based chemotherapy as standard treatment; however, the benefit is small, and this is probably attributed to either intrinsic or developed chemoresistance in the majority of cancer cells. As GEM has an irreplaceable role in the treatment of PDAC, understanding the mechanism underlying GEM resistance is extremely important. Our study demonstrated that DDB1 is associated with the key enzyme dCK, which is required for GEM to exert its antitumor activity. Additionally, DDB1 itself affects the cell proliferation rate in vitro and in xenograft models of PDAC. Furthermore, DDB1 is an independent prognostic factor that can be used to predict the prognosis for patients with PDAC.

Despite its main role in nucleotide excision repair, cell proliferation and apoptosis, DDB1 is also involved in many signaling pathways related to carcinogenesis and multiple oncoproteins [18,19]. The CRL4 ubiquitin E3 ligase complex inhibits mammalian target of rapamycin (mTOR) signaling through a DDB1-binding WD40 protein and is involved in myc degradation through the proteasome [20,21]. DDB1 also participates in drug metabolism in cancer therapy and is associated with chemoresistance. Nucleolar sirtuin 7 (SIRT7) promotes DDB1 deacetylation, resulting in a decrease in DDB1-CUL4 activity and contributing to apoptosis in cells treated with 5-fluorouracil; in addition, sensitivity to cisplatin treatment could be stimulated by silencing DDB1 [15,22].

Studies have demonstrated that the relationship between changes in morphology and EMT induction in PDAC cells develops at a very early stage and influences cell motility as well as tumor chemoresistance [23,24]. Our previous study showed that glutathione peroxidase-1 (GPX1) could inhibit EMT and GEM resistance by regulating the AKT/GSK-3β/SNAI1 signaling axis in PDAC [16]. We found decreased mesenchymal markers and increased epithelial markers in DDB1 knockdown PDAC cells compared to normal PDAC cells. Numerous studies have suggested that EMT is pivotal for PDAC invasion and metastasis, but genetically engineered mouse models with SNAI1 deletion failed to alter the progression of PDAC [25]. Overexpression of DDB1 and CUL4 associated factor 4 like 2 (DCAF4L2) could promote EMT by activating the nuclear factor kappa-B (NF-κB) signaling pathway, but the relationship between EMT and DDB1 as a single unit has not been explored [26]. Our study initially demonstrated that *DDB1*, as an oncogene, could enhance EMT by upregulating *SNAI1* and *ZEB1*, which are 2 important transcription factors in the EMT program. It is possible that DDB1 serves as a deubiquitinase of SNAI1, and DDB1 knockdown might lead to SNAI1 destabilization and EMT suppression; DDB1, a component of the E3 ubiquitin-protein ligase complex, could mediate similar ubiquitination and degrade proteasomes [27]. ZEB1, a regulator of the DNA damage response, is associated with USP7 and could enhance its ability to deubiquitylate, which results in the promotion of radioresistance. ZEB1 might accelerate the deubiquitylating process of DDB1, but it still requires more experiments to confirm whether it interacts with DDB1 through the homologous recombination pathway [28].

The results of the present study suggest that DDB1 knockdown can increase dCK expression in PDAC cells. dCK mediates the rate-limiting catabolic step in the process of GEM activation, which is an independent and strong prognostic factor in patients with PDAC [29]. A important GEM-resistant pathway, cysteine-rich 61 (CYR61)/ cellular communication network factor 1 (CCN1), could downregulate dCK and induce connective tissue growth factors in vitro and in vivo to create a desmoplastic reaction and chemoresistance [30]. dCK could also participate in DNA damage and repair induced by ionizing radiation because it interacts with cyclin-dependent kinase 1 and is required for the G2/M checkpoint, and dCK contributes to resistance to radiotherapy [31,32,33]. We found that dCK was also associated with SNAI1 expression at both the RNA and protein levels, and it is possible that DDB1 induces GEM resistance through novel mechanisms. Hu et al. found that decreased dCK expression could worsen GEM resistance by promoting the master regulator of redox homeostasis NF-E2 p45-related factor 2 (NRF2) and by forming a feedback loop [17]. The decreased tumor sizes and low Ki-67 expression in tumor xenograft models further indicated that DDB1 regulates GEM metabolism by affecting dCK, which expands the size of the dNTP pools. There are several studies relating GEM resistance and EMT phenotypes as well as their regulation through cancer-related pathways and GEM metabolic enzymes. The participation of the hypoxia-inducible factor 1α (HIF-1α)/signal transducer and activator of transcription 3 (STAT3) signaling pathway was investigated in GEM-resistant pancreatic cancer cells by mediating the expression of ZEB1 and TWIST1, thereby regulating GEM metabolic enzymes and contributing to GEM-resensitized cell death induction [34]. F-Box and WD repeat domain containing 7 (FBXW7) functions in phosphorylation-dependent ubiquitination; it is also a ligase of E3 ubiquitin, and was proven to be a target for inducing the expression of GEM metabolic enzymes to improve GEM efficacy [35]. However, although DDB1 has a similar ubiquitin function as FBXW7, the underlying mechanisms of DDB1 and dCK still require further exploration.

## 4. Materials and Methods

### 4.1. Cell Culture

The human pancreatic cancer cell lines MiaPaCa-2 and PANC-1 were obtained from the American Type Culture Collection (ATCC, USA) and verified by DNA fingerprinting. The cells were cultured in a humidified incubator at 37 °C with 5% CO_2_ and tested for mycoplasma contamination by PCR every 3 months. The culture medium for MiaPaCa-2, PANC-1 and HPDE cells strictly followed protocols described previously [16,36].

### 4.2. IHC

TMAs were obtained from patients histopathologically and clinically diagnosed with PDAC at the Fudan University Shanghai Cancer Center (FUSCC) from 2010 to 2012. Informed consent was obtained from each patient, and all experiments were performed with the approval of the Clinical Research Ethics Committee of FUSCC (ethical code: 050432-4-1212B). Two experienced pathologists participated in the disease diagnoses and immunohistochemical staining scoring. Anti-DDB-1 (1:100; Abcam, UK, ab109027), anti-Ki-67 (1:400; Cell Signaling Technology (CST), USA, #12202S) and anti-dCK (1:1000; Abcam, ab151966) were used as antibodies to detect protein expression levels based on methods previously described [17]. Immunohistochemical staining scores were generated by multiplying the percentage of stained positive cells (0, <5%; 1, 5-25%; 2, 25-50%; 3, 50-75%; and 4, >75%) and the staining intensity (0, negative; 1, weak; 2, moderate; and 3, strong), which classified the expression levels as follows: negative (0, -), weak (1–3, +), moderate (4–6, ++) and strong (>6, +++). Patients could be divided into 2 groups based on their scores (-/+ as low expression and ++/+++ as high expression).

### 4.3. Plasmids

The 19 base pairs (bp) targeted against DDB1 were TATCACAATGGTGACAAAT and ACTCAATAAAGTCATCAAA. The lentiviral cloning vector pLKO.1 TRC (Addgene, USA) was later ligated with the shRNA oligos based on a standard procedure [37]. Silencing lentivirus particles were produced by cotransfecting lentivirus constructs with pMD2.G and psPAX2 in a 4:3:1 ratio and then added to the HEK-293T cells. The pLKO.1 scrambled-shRNA was designed as a control plasmid.

### 4.4. Cell Viability Assay

Cell proliferation and cell cytotoxicity were determined through cell viability assays using a Cell Counting Kit-8 (CCK-8; Dojindo, Japan) and were conducted as previously described [17]. The IC_50_ value was calculated through a nonlinear least-squares curve that fit to the dose-response curves.

### 4.5. Flow Cytometry

Cells were stained with a Annexin V PE Apoptosis Detection Kit (BD, La Jolla, CA, USA) according to the manufacturer’s instructions. The percentage of apoptotic cells was later analyzed by a FACSCalibur flow cytometer.

### 4.6. Wound Healing Assay and Transwell Migration Assays

Cells were digested and seeded into 6-well plates to form a confluent monolayer for 24 h and replaced with serum-free culture medium for starvation overnight. One wound per well was conducted with a 10-µL tip and washed twice with PBS before live cell imaging was taken at 0 h and 24 h. The migration speed was determined by measuring the wound areas in triplicate for each image (200x, Olympus, Japan). Migration assays were performed as described previously [16]. The cells were plated in 24-well transwell chambers (Corning, USA). The cells on the top surface were removed with a cotton swab, and the cells on the lower surface of the chamber were washed, fixated, stained and photographed (200x, Olympus).

### 4.7. RNA Isolation and Quantitative Real-Time PCR

Total RNA was extracted using TRIzol Reagent (Invitrogen, USA). Reverse transcription was conducted using a TaKaRa PrimeScript RT Reagent Kit (TaKaRa, Japan). Forty-five paired PDAC samples were extracted from histopathologically and clinically diagnosed patients at FUSCC. The expression of candidate genes was determined using an ABI 7900HT Real-Time PCR System (Applied Biosystems, USA). The primer sequences used in this study are presented in Supplement material Table S2.

### 4.8. Western Blotting

Western blotting was performed as previously described [38]. The antibodies used in our study were against β-actin (1:5000; Proteintech, USA, 60008-1-Ig), DDB1(1:50,000; Abcam, ab109027), dCK (1:10,000; Abcam, ab151966), SNAI1 (1:1000; CST, #3879S), ZEB1 (1:1000; CST, #3396S) and VIMENTIN (1:1000; CST, #5741S).

### 4.9. Colony Formation Assay

After digestion and counting, 500 cells were seeded and incubated in 6-cm cell culture dishes for 14 days. Cells were fixed with 4% paraformaldehyde and stained with a 0.1% crystal violet solution (Sigma, USA). Colonies with more than 50 cells were counted under a light microscope.

### 4.10. RNA Sequencing

Total RNA was isolated from scrambled-shRNA- and DDB1-shRNA-transfected MiaPaCa-2 cells using TRIzol Reagent (Invitrogen). The cell lines were analyzed in triplicate. RNA sequencing was carried out using an Illumina HiSeq 4000 Sequencing System. Analysis of the fragments per kilobase of exon per million mapped reads for each gene was conducted after data preprocessing and collection.

### 4.11. Animal Model

BALB/c-nu mice aged 4 to 6 weeks (Shanghai SLAC Laboratory Animal, China) were housed in sterile and filter-capped cages. Approximately 3 × 10^6^ stably expressing sh-DDB1 and scrambled-shRNA cells in 100 μL PBS were injected subcutaneously into the right flanks of the mice. These mice were then divided into GEM-treated and PBS-treated subgroups for each cell line (*n* = 4 each group). After tumor formation in 2 weeks, the size of the xenograft was measured according to the tumor length and width once a week, and the tumor volume was calculated by the following formula: length × width^2^ × 0.5. GEM (20 mg/kg) was injected intraperitoneally twice a week. All tumor specimens were surgically removed after 6 weeks of tumor implantation and processed with 4% paraformaldehyde before being sectioned into tissue slices for immunohistochemical staining. All procedures were performed strictly followed the protocol approved by the Committee on the Ethics of Animal Experiments of Fudan University (ethical code: 2016 0 815 A148).

### 4.12. Statistical Analysis

All experimental data are presented as the mean ± SD and were repeated at least 3 times and analyzed by SPSS 22.0 software (Abbott Laboratories, USA). Student’s *t* test and one-way ANOVA were used to analyze the data between groups. *χ*^2^ or Fisher’s exact test was employed for the correlation analysis. A *p* < 0.05 was considered statistically significant.

## 5. Conclusions

In conclusion, we showed that DDB1 is highly expressed in PDAC and is associated with poor prognosis. DDB1 promotes cell proliferation in vitro and in xenograft models, reduces GEM-induced cell apoptosis and drives EMT by upregulating SNAI1, ZEB1 and VIMENTIN. We also found that DDB1 expression is negatively correlated with dCK, which is indispensable for the maintenance of the active form of GEM. Therefore, we believe that DDB1 might have the potential to serve as a novel predictive and therapeutic target for PDAC.

## Figures and Tables

**Table 1:** Relationship between DDB1 expression and patient clinicopathological features of PDAC.

		DDB1 Expression		
Features	Total (*n* = 147)	Low (*n* = 34)	High (*n* = 113)	*p*
**Age (years)**				0.136
≤60	70	20	50	
>60	77	14	63	
**Sex**				0.477
Male	83	21	62	
Female	64	13	51	
**AJCC stage**				0.246
I-IIA	78	21	57	
IIB-III	69	13	56	
**Grade**				0.888
High/moderate	88	20	68	
Low	59	14	45	
**Tumor size (cm)**				0.646
<4	99	24	75	
≥4	48	10	38	
**Vascular invasion**				0.994
No	121	28	93	
Yes	26	6	20	
**CA19-9≥37 U/mL**				0.678
No	35	9	26	
Yes	112	25	87	

**Table 2:** Univariate and multivariate Cox regression of overall survival for PDAC patients.

Features	N	Univariable Analysis		Multivariable Analysis	
		HR (95% CI)	*p*	HR (95% CI)	*p*
**All**					
**Age (years)**					
>60	77	1.134 (0.805–1.596)	0.472		
≤60	70				
**Sex**					
Female	64	1.184 (0.836–1.677)	0.341		
Male	83				
**AJCC stage**					
IIB-III	69	1.883 (1.334–2.660)	<0.001	1.846 (1.307–2.609)	0.001
I-IIA	78				
**Grade**					
Low	59	1.223 (0.862–1.735)	0.259		
High/moderate	88				
**Tumor size (cm)**					
≥4	48	1.496 (1.038–2.157)	0.031	1.522 (1.054–2.197)	0.025
<4	99				
**Vascular emboli**					
Yes	26	1.248 (0.800–1.945)	0.329		
No	121				
**DDB1 expression**					
High	113	1.990 (1.288–3.076)	0.002	1.909 (1.235–2.950)	0.004
Low	34				
**CA19-9≥37 U/mL**					
Yes	112	0.933 (0.626–1.390)	0.732		
No	35				

HR, hazard ratios; CI, confidential intervals.

## Supplementary Materials

The following are available online at https://www.mdpi.com/2072-6694/11/12/1998/s1, Figure S1: Western blotting data, Table S1: The IC_50_ values for GEM in PDAC cells, Table S2. Primer sequences used in the study, Table S3. Intensity ratio of Western blotting in Figure 2A,B,I and Figure 4F.
