# Supplementary material for: Role of Damage DNA-Binding Protein 1 in Pancreatic Cancer Progression and Chemoresistance
Source: Cancers (Basel). 2019 Dec 12;11(12):1998. doi: 10.3390/cancers11121998 (PMC6966444; doi:10.3390/cancers11121998)
Supplement: Supplementary file 1 [file cancers-11-01998-s001.pdf]

cancers MDPI

Supplementary materials:

Table S1. The IC_{50} values for GEM in PDAC cells.

Cell lines	IC_{50}	Cell lines	IC_{50}
MiaPaCa-2-scrambled	1.853±0.103 μM	PANC-1-scrambled	10.338±0.830 μM
MiaPaCa-2-shDDB1-A	0.403±0.024 μM	PANC-1-shDDB1-A	2.831±0.312 μM
MiaPaCa-2-shDDB1-B	0.366±0.048 μM	PANC-1-shDDB1-B	3.379±0.194 μM

Table S2. Primer sequences used in the study.

Gene Name	Direction	Primer Sequence (5'-3')
DDB1	Forward	TGCGGTCTGACCCTAATCGT
	Reverse	GACCAACCTCACCGATGCTG
dCK	Forward	CAAGACTGGCATGACTGGATGAA
	Reverse	GGCACCTCTTGAAGATAATCGAAG
SNAI1	Forward	CCTTCTCTAGGCCCTGGCTGCTAC
	Reverse	GACATCTGAGTGGGTCTGGAGGTGG
ZEB1	Forward	GCATACAGAACCCAACTTGAACG
	Reverse	TGGGCATTCATATGGCTTCTCTCC
VIMENTIN	Forward	CCCTTGACATTGAGATTGCCACCT
	Reverse	GTGGGTATCAACCAGAGGGAGTGA
β-actin	Forward	AGAGCTACGAGCTGCCTGAC
	Reverse	AGCACTGTGTTGGCGTACAG

Table S3. Intensity ratio of Western blotting in Figure 2A, 2B, 2I and 4F.

**Fig 2A**	PANC-1	Capan-1	BXPC-3	CFPAC-1	MiaPaCa-2	SW1990	HPDE
Intensity ratio of DDB1	4.00	3.62	3.54	2.20	4.63	1.28	1.00
Intensity ratio of β-actin	1.10	1.08	1.07	1.00	0.99	0.92	1.00
**Fig 2B**		MiaPaCa-2			PANC-1		
	Scramble	shDDB1-A	shDDB1-B	Scramble	shDDB1-A	shDDB1-B	
Intensity ratio of DDB1	1.00	0.32	0.36	1.00	0.26	0.32	
Intensity ratio of β-actin	1.00	0.94	0.96	1.00	1.00	0.93	
**Fig 2I**		MiaPaCa-2			PANC-1		
	Scramble	shDDB1-A	shDDB1-B	Scramble	shDDB1-A	shDDB1-B	
Intensity ratio of ZEB1	1.00	0.40	0.10	1.00	0.15	0.26	
Intensity ratio of VIMENTIN	1.00	0.40	0.25	1.00	0.43	0.49	
Intensity ratio of SNAI1	1.00	0.11	0.18	1.00	0.21	0.09	
Intensity ratio of β-actin	1.00	1.01	1.08	1.00	0.92	0.99	
**Fig 4F**		MiaPaCa-2			PANC-1		
	Scramble	shDDB1-A	shDDB1-B	Scramble	shDDB1-A	shDDB1-B	
Intensity ratio of dCK	1.00	2.02	1.76	1.00	1.65	1.59	
Intensity ratio of β-actin	1.00	0.97	0.89	1.00	0.86	0.98	

cancers

Figure S1. Western blotting data.

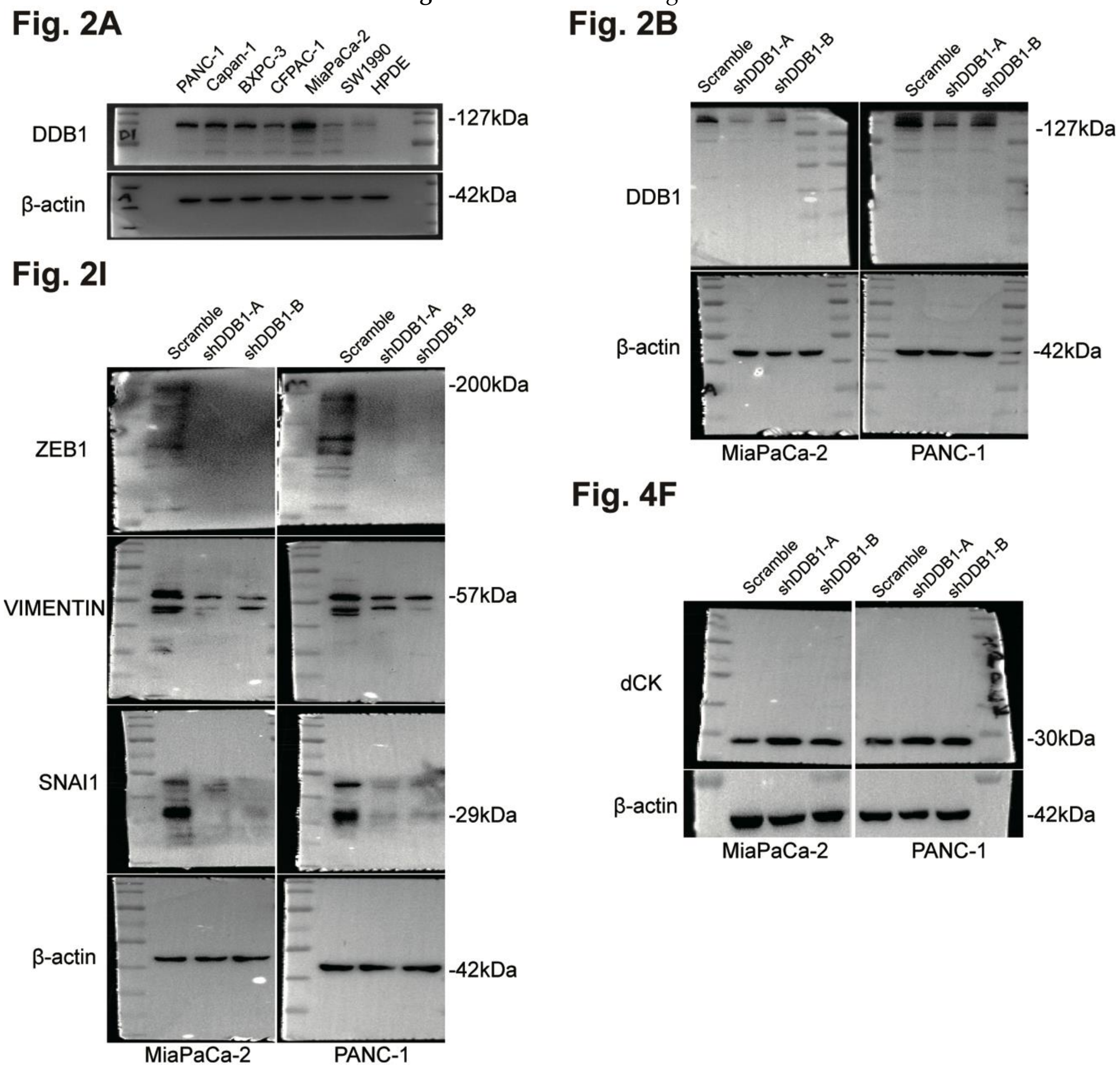

© 2019 by the authors. Submitted for possible open access publication under the terms and conditions of the Creative Commons Attribution (CC BY) license (http://creativecommons.org/licenses/by/4.0/).
